# Reactivation of Human Herpesvirus-6 in Natalizumab Treated Multiple Sclerosis Patients

**DOI:** 10.1371/journal.pone.0002028

**Published:** 2008-04-30

**Authors:** Karen Yao, Susan Gagnon, Nahid Akhyani, Elizabeth Williams, Julie Fotheringham, Elliot Frohman, Olaf Stuve, Nancy Monson, Michael K. Racke, Steven Jacobson

**Affiliations:** 1 Viral Immunology Section, National Institutes of Health, Bethesda, Maryland, United States of America; 2 Department of Neurology and the Center for Immunology, The University of Texas Southwestern Medical Center, Dallas, Texas, United States of America; 3 Department of Biology, Johns Hopkins University, Baltimore, Maryland, United States of America; Institut Pasteur Korea, Republic of Korea

## Abstract

The α_4_ integrin antagonist natalizumab was shown to be effective in patients with immune-mediated disorders but was unexpectedly associated with JC polyomavirus associated progressive multifocal leukoencephalopathy (PML) in two multiple sclerosis (MS) and one Crohn's disease patients. Impaired immune surveillance due to natalizumab treatment may have contributed to the JCV reactivation. As HHV-6 has been suggested to play a role in MS, we asked whether this virus could also have been reactivated during natalizumab therapy. Matched sera and CSF from a limited set of MS patients treated with and without natalizumab were examined for evidence of HHV-6. In addition, we also superinfected a persistent JC virus infected glial cell with HHV-6A to determine if JC virus can be increased. Elevated serum HHV6 IgG and HHV-6A DNA was detected in the CSF of a subset of patients but not controls. We confirmed that superinfection with HHV-6 of a JC virus infected glial cells increased expression of JCV. These results support the hypothesis that treatment with natalizumab may be associated with reduced immune surveillance resulting in reactivation of viruses associated with MS pathogenesis.

## Introduction

Multiple sclerosis (MS) is an immune mediated demyelinating disorder of the central nervous system (CNS) characterized by inflammatory lesions in the brain and spinal cord that result in varying degrees of neurological impairment. Self-reactive lymphocytes are believed to play a role in the pathogenesis of MS [1]. To access the central nervous system (CNS), cells must cross the blood-brain barrier by binding to adhesion molecules present on vascular endothelial cells. Thus, inhibiting access of these inflammatory cells to the CNS by interference with molecules involved in vascular adhesion is an attractive therapeutic target. Natalizumab, is a humanized monoclonal antibody against the α_4_ subunit of α_4_β_1_ and α_4_β_7_ integrins, which are molecules involved in cell motility through interaction with ligands in the extracellular matrix. Natalizumab blocks the interaction of these molecules with their receptors, vascular-cell adhesion molecule 1 (VCAM-1) and mucosal addressin-cell adhesion molecule 1, present on the vascular endothelium resulting in decreased migration of peripheral inflammatory cells into the target tissues [2], [3].

While natalizumab was shown to be clinically effective in patients with relapsing-remitting MS in two phase III clinical trials (US FDA. Natalizumab (marketed as Tysabri) Information. http://www.fda.gov/cder/drug/infopage/natalizumab/default.htm (2005)), it was unexpectedly associated with a rare neurological complication in two patients with MS and one with Crohn's disease who developed progressive multifocal leukoencephalopathy (PML) [4], [5], [6]. Two patients subsequently died [7], [8] and natalizumab was voluntarily withdrawn from the market. PML is a CNS opportunistic infection caused by reactivation of a clinically latent JC polyomavirus that infects and destroys oligodendrocytes leading to multifocal areas of demyelination and associated neurologic dysfunction [9]. PML invariably occurs in the context of impaired cell-mediated immunity most frequently observed in individuals with compromised immune systems. One mechanism suggested to explain the relationship between natalizumab treatment and development of PML is that by blocking α4 integrin and thus decreasing lymphocyte trafficking to the brain [10], the normal immune surveillance in the brain was reduced, allowing the reactivation of latent viruses present in the nervous system [11]. In support of this hypothesis, we recently reported that the CD4∶CD8 ratio in the CSF of MS patients treated with natalizumab was reduced to levels similar to that observed in HIV infection [12].

If impaired immune surveillance in the brain following natalizumab treatment is associated with JCV reactivation, there is no reason to believe, *a priori*, that such a mechanism would be specific for JCV and thus such impaired surveillance could result in the reactivation of other latent CNS viruses. Human herpesvirus-6 (HHV-6) is a pleiotropic β-herpesvirus that has been shown to infect cells of the CNS [13], [14]. Two variants of this virus have been described[15]. HHV-6B is the etiologic agent of the childhood disease exanthem subitum (roseola)[16] while the HHV-6A variant is considered more neurotropic with distinct host cell tropisms[17]. Like JCV, HHV-6 establishes latency in the host and can be reactivated under conditions of immunosuppression. Moreover, there is a large body of literature that has postulated a role for HHV-6 in the pathogenesis of MS [18], [19], [20], [21], [22], [23], [24], [25], [26], [27]. Studies have consistently reported increased antibody responses to HHV-6 proteins in MS patients, demonstrated the presence of HHV-6A DNA in serum (indicative of a productive infection), particularly during clinical exacerbations, and pathologically HHV-6 is more often detected in regions of MS plaques compared to normal appearing white matter. Interestingly, HHV-6 has also been associated with PML. HHV-6 and JCV have been detected in white matter both within and surrounding PML lesions and in intralesional oligodendrocytes [28].

Based on the previously established associations of HHV-6 with MS and the unexpected reactivation of latent virus in natalizumab-treated patients, we asked whether HHV-6 might also be reactivated in MS patients treated with natalizumab. We examined sera and matched CSF from a cohort of MS patients who received natalizumab compared to MS patients with clinically definite MS not treated with natalizumab. Detection of cell-free HHV-6A DNA sequences (the more neurotropic HHV-6 variant more often associated with MS) in CSF and increased frequency of serum HHV-6 specific antibodies in natalizumab treated MS patients suggest that reactivation of HHV-6 may also be associated with natalizumab therapy. While the relationship of natalizumab therapy with HHV-6 and JCV-PML is unclear, we also demonstrate that, in vitro, HHV-6 significantly augments the replication of JCV. These results suggest a possible mechanism whereby natalizumab treatment may reactivate latent viruses of the CNS (such as HHV-6) that can subsequently induce the replication of other viruses (such as JCV) that have been associated with catastrophic outcomes (PML) in a small subset of patients.

## Results

### Detection of HHV-6 DNA in CSF from natalizumab-treated MS patients

**Figure 1:**
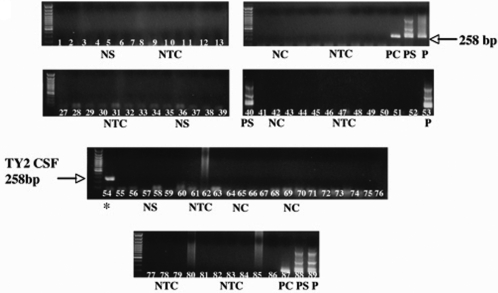
Detection of HHV-6A DNA in CSF of natalizumab-treated MS patients. Representative figure of nested PCR detection for HHV-6 in CSF and sera of UT-MS patients. Lane 54  =  PCR detection in CSF sample of a natalizumab treated patient TY2. NS  =  known negative serum control; NTC  =  no template control; NC  =  known CSF control; PC  =  CSF positive control; PS  =  known serum positive control; P  =  positive control (HHV-6B Z29 infected SupT-1cells).

Using nested PCR for the HHV-6 U57 gene, cell-free viral DNA was detected in 2 out of 23 natalizumab-treated patients at UT Southwestern (Figure 1). Sample TY2 was positive on the initial screen of CSF from natalizumab treated MS patients while sample TY3 was positive from a follow-up CSF sample obtained 4 months after the first lumbar puncture that was HHV-6 negative. In contrast, HHV-6 viral sequences were not detected in 16 samples from non-natalizumab treated MS patients collected at the same center, consistent with our inability to detect HHV-6 DNA in CSF from 31 NIH MS patients and 16 control patients with other neurological disorders (as summarized in table 1). The number of natalizumab-treated patients positive for HHV-6 DNA in CSF was obviously too small to achieve statistical significance in relation to non-natlizumab treated MS patients from the same center (Table 1). However, given the limited number of patients and our experience with the detection of HHV-6 in CSF (Table 1), detection of low levels of HHV-6 in the CSF of MS patients treated with natalizumab was unexpected.

**Table 1:** UT Texas Natalizumab treated and Untreated MS Patients.

Patient	Age^1^	Gender	Diagnosis	EDSS^2^	Clinical Trial
**Natalizumab Treated Patients**					
TY1	41	Male	RRMS/1998	1.5	N/A^3^
TY2	48	Female	RRMS/1990	4.5	AFFIRM^4^
TY3	51	Female	RRMS/2001	0	AFFIRM
TY4	46	Female	RRMS/1994	4.5	AFFIRM
TY5	37	Female	RRMS/1992	0	AFFIRM
TY6	53	Female	RRMS/2002	4	AFFIRM
TY7	38	Female	RRMS/1995	3.5	AFFIRM
TY8	42	Female	RRMS/1999	1.5	SENTINEL^5^
TY9	46	Female	RRMS/1999	3.5	SENTINEL
TY10	50	Female	RRMS/1995	2.5	SENTINEL
TY11	53	Female	RRMS/1994	6	SENTINEL
TY12	52	Female	RRMS/1989	2.5	SENTINEL
TY13	35	Female	RRMS/1992	1.5	SENTINEL
TY14	51	Female	RRMS/1999	1.5	SENTINEL
TY15	46	Male	RRMS/1996	4	SENTINEL
TY16	31	Female	RRMS/2000	1.5	SENTINEL
TY17	35	Female	RRMS/1992	1.5	SENTINEL
TY18	45	Female	RRMS/1996	3.5	SENTINEL
TY19	45	Female	RRMS/1999	2.5	SENTINEL
TY20	31	Female	RRMS/1999	2	SENTINEL
TY21	43	Female	RRMS/2001	2	SENTINEL
TY22	54	Male	RRMS/2001	1.5	SENTINEL
TY23	51	Female	RRMS/2001	3.5	SENTINEL
**Untreated or Non-Natalizumab Treated Patients**					
UTMS24	44	Female	PPMS/2000	6	none
UTMS25	26	Female	RRMS/2005	1.5	none
UTMS26	60	Female	PPMS/1999	6	none
UTMS27	58	Female	PPMS/1996	ND	none
UTMS28	63	Male	SPMS/1977	ND	cellcept and avonex
UTMS29	58	Male	PPMS/2000	6	none
UTMS30	48	Male	PPMS/2000	ND	none
UTMS31	62	Male	CIS-ON/2005	ND	none
UTMS32	61	Female	PPMS/2001	3	none
UTMS33	49	Male	PPMS/2002	6	none
UTMS34	35	Female	RRMS/2001	ND	betaseron
UTMS35	66	Male	PPMS/1991	4	none
UTMS36	37	Male	PPMS/2004	3.5	none
UTMS37	42	Male	PPMS/2000	4.5	none
UTMS38	32	Female	RRMS/2002	ND	none
UTMS39	53	Female	SPMS/1994	ND	avonex
UTMS40	47	Male	PPMS/2000	3.5	none

1 Age assessed at time of enrollment in this study.

2 EDSS assessed at time of enrollment in this study.

3 N/A  =  Not applicable. Patient started natalizumab after initial approval by the Food and Drug Administration.

4 AFFIRM  =  Natalizumab safety and efficacy in relapsing remitting multiple sclerosis.

5 SENTINEL  =  The safety and efficacy of natalizumab in combination with interferon beta-1a in patients with relapsing-remitting multiple sclerosis RRMS  =  relapsing-remitting multiple sclerosis; PPMS  =  primary progressive multiple sclerosis; SPMS  =  secondary progressive multiple sclerosis; CIS-ON  =  clinically isolated syndrome/optic neuritis; ND  =  not determined.

It was also critical to determine which variant of HHV-6 was detected in CSF of natalizumab-treated MS patients. DNA sequencing of the amplified HHV-6 U57 gene (data not shown) from both CSF-positive natalizumab-treated MS patients showed 99% identity to the HHV-6A U1102 variant as represented by the results in figure 1 for patient TY2 (Figure 1). These results confirm the observation that amplifiable HHV-6 sequences were detected in CSF of natalizumab-treated MS patients and subtyped as HHV-6A, the variant more frequently associated with MS [18], [22], [24].

### Increased anti-HHV-6 IgG in serum and CSF of MS patients

**Figure 2:**
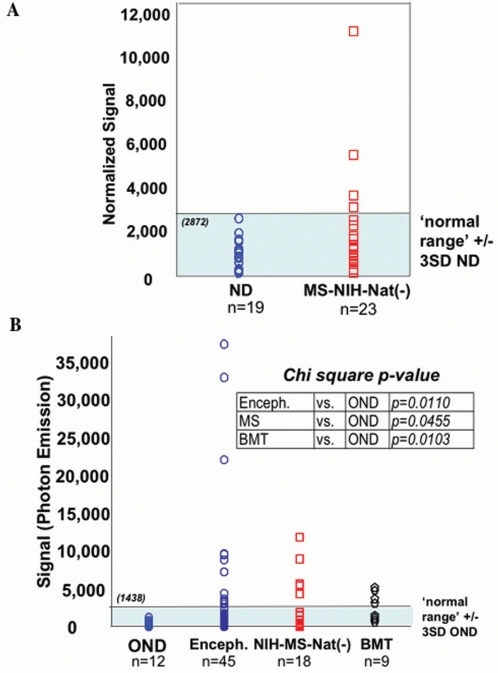
Detection of anti-HHV-6 IgG in serum and CSF of MS patients. (A) HHV-6 antibody in sera of 19 healthy controls (ND) and 23 NIH MS patients without natalizumab treatment. Normalized signal calculated as reactivity on [(HHV-6B infected lysate - uninfected cells)/total human IgG]. Normal range is 3 standard deviations (SD) above the mean normalized signal (2872) of ND. (B) Comparison of HHV6 reactivity in CSF from other neurologic controls (OND), encephalitis (Enceph.), untreated NIH MS patients (NIH-MS-Nat (−)) and BMT recipients with neurologic complications. HHV6 reactivity measured as photon emission on HHV-6B virus infected lysate minus emission on uninfected cells. Normal range is 3SD above mean OND signal (1438).

Previous work from our group demonstrated, by ELISA, that there was no statistically significant difference in the amount of anti-HHV-6 IgG in the serum of MS patients versus normal donors or other inflammatory and other neurological disease controls [24]. Here, we examined sera from 19 normal donors and 23 untreated MS patients (a subset of the NIH cohort) for serum IgG against HHV-6 using the HHV-6 ECL assay. Values obtained with this assay were normalized to total serum IgG for each patient. As predicted, figure 2A shows no statistically significant difference in anti-HHV-6 serum IgG between the two groups although several MS patients demonstrated levels of serum IgG in excess of the empirically defined “normal range”, operationally defined as 3 standard deviations above the mean for all normal sera.

The presence of anti-HHV-6 IgG in CSF has been reported in MS, particularly during exacerbations [29], [30]. We compared anti-HHV-6 IgG levels in the CSF between other neurologic disease controls (OND, Table S1), patients with suspected viral encephalitis, untreated MS patients (a subset of the NIH cohort), and patients with infectious complications post-bone marrow transplant (BMT) [31]. ‘Normal range’ for HHV-6 IgG in CSF was defined as 3 standard deviations above the mean for CSF of patients with OND (Figure 2B). As positive controls, CSF from patients with encephalitis and neurologic disease post (BMT) were used since subsets of these patients have been reported to be reactive for HHV-6 [31], [32], [33]. This observation was confirmed using our novel HHV-6 ECL assay in CSF from the above patient cohorts, demonstrating statistically elevated levels of HHV-6 IgG in post-BMT CSF compared to control OND CSF (Figure 2B). Similarly, untreated MS patients exhibited significantly higher levels of anti-HHV-6 IgG in the CSF compared to control OND CSF, consistent with previous results demonstrating elevated HHV-6 antibody titers in CSF of MS patients [24]. Collectively, these results help to characterize the HHV-6 ECL assay as a reliable and robust method for the detection of HHV-6 IgG in serum and CSF.

### Increased anti-HHV-6 IgG in serum and CSF of natalizumab-treated MS patients

**Figure 3:**
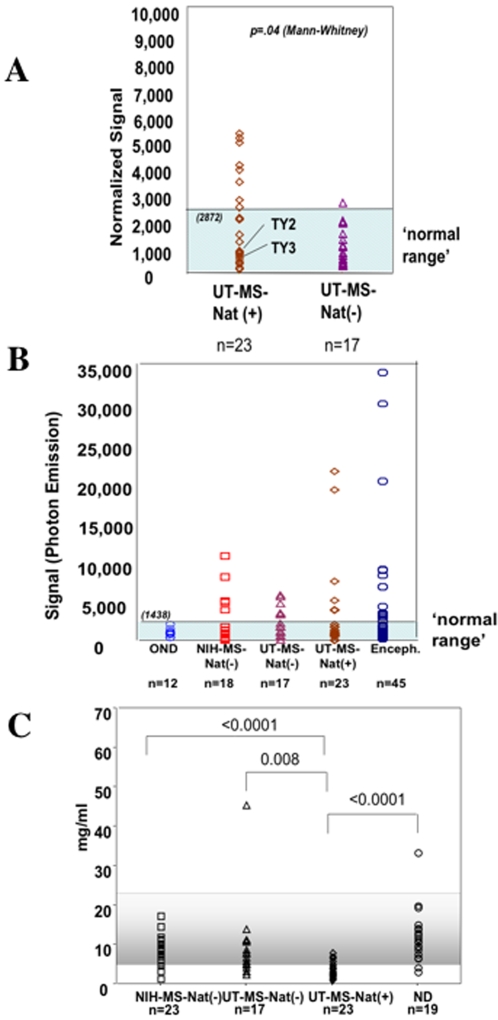
Natalizumab-treated MS patients have increased levels of serum IgG against HHV-6. (A) HHV-6 reactivity in sera of treated University of Texas (UT) MS patients (UT-MS-Nat (+)) or not treated with natalizumab (UT-MS-Nat (−)). Normal range same as in figure 3. (B) HHV-6 antibody levels in CSF of other neurologic controls (OND), encephalitis (Enceph.), NIH MS patients (NIH-MS-Nat-), MS patients from UT not-treated or treated with natalizumab (UT-MS-Nat (−) and UT-MS-Nat (+)). Normal range is defined as described above. (C) Total human IgG values (mg/ml) in sera of MS cohorts and healthy controls (ND). Significance values calculated using Mann-Whitney.

Based on the earlier reports of HHV-6 with both MS and PML [28], and the results in this study detecting HHV-6A DNA sequences in a subset of natalizumab treated MS patients (Figure 1), we investigated whether differential antibody responses between MS patients treated with and without natalizumab could be demonstrated. HHV-6 IgG levels were measured in serum from all 23 natalizumab-treated MS patients and 17 MS patients from the same center (UT Southwestern) who did not receive natalizumab (Table 1). As shown in figure 3A, natalizumab-treated MS patients had significantly higher levels of serum HHV-6 IgG than MS patients not treated with natalizumab. Serum HHV-6 IgG levels from the UT Southwestern non-natalizumab treated MS patients were similar to the non-natalizumab treated NIH MS cohort (Figure 3A; p =  .78). Analysis of HHV-6 IgG in CSF samples was also performed. As we have shown that CSF HHV-6 IgG was elevated in non-natalizumab treated MS groups compared to CSF from patients with OND (Figure 3B), there was still a trend towards higher levels of CSF HHV-6 IgG in a subset of natalizumab treated MS patients (Figure 3B) although this did not reach statistical significance in this limited cohort. In addition, the ratio of HHV6 IgG in the CSF versus serum was elevated in a subset of UT natalizumab treated MS patients 1.4±0.6 (TY2, TY3, TY5, TY8, TY29, and TY21) compared to the mean value of UT non-natalizumab treated patients (0.3±0.2) or the NIH-untreated RRMS patients (0.1±0.1). Of particular interest is the observation that the HHV-6 CSF/serum ratio for TY2 and TY3 who were shown to contain HHV-6 DNA sequences in CSF (Figure 1) was 0.9 and 1.3, respectively.

As the quantitation of serum HHV-6 IgG is based on normalization to total serum IgG, it became evident that natalizumab-treated MS patients had lower levels of total serum IgG than non-natalizumab treated MS patients from the same center (Figure 3C). The hatched area denotes the normal range of total serum IgG (5.5–22 mg/ml) as per the manufacturer's instructions. Normal donors and untreated MS patients from the NIH-cohort fell within this range with no differences demonstrated between these two groups (Figure 3C). While the non-natalizumab treated MS cohort from UT-Texas had lower total serum IgG, the natalizumab-treated MS cohort had the lowest total serum IgG as (Figure 3C). Decreased serum IgG levels are characteristic of immunosuppression and such decreases maybe associated with reactivation of several viruses including HHV-6 [34], [35], [36].

### Up-regulation of JCV by HHV-6

**Figure 4:**
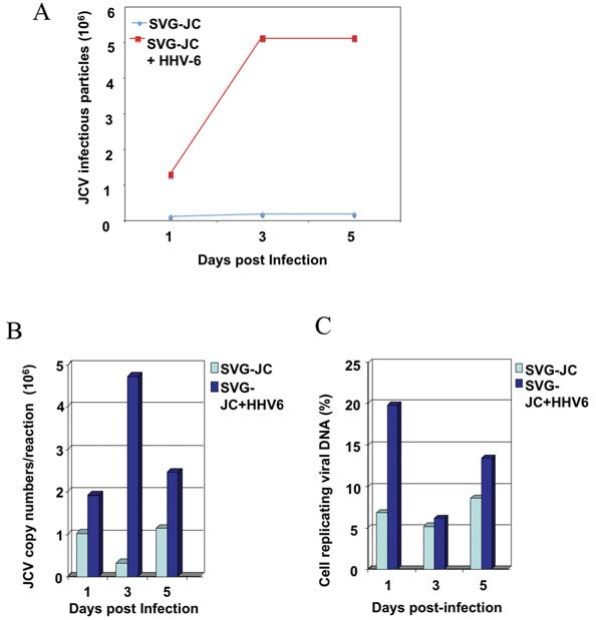
Superinfection of human glial (SVG-JC cells) with HHV-6 can augment JCV replication. (A) Hemagglutination assay for quantitation of JCV infectious particles in JC virus persistently infected SVG cells (SVG-JC) or SVG-JC cells super infected with HHV-6A. (B) Quantitative real-time PCR detection of JCV DNA in JC virus persistently infected SVG cells (SVG-JC) or SVG-JC cells super infected with HHV-6A. (C) Quantitation of *in situ* hybridization studies demonstrating percent of cells with replicating JCV viral DNA in SVG-JC or SVG-JC cell superinfected with HHV-6A.

Since reduced immune surveillance due to natalizumab treatment may reactivate latent viruses of the CNS such as HHV-6 it was of interest to determine if HHV-6 could be associated with increased expression of JC virus. To investigate whether HHV-6 could upregulate JCV expression, an SV40-transformed human glial cell line (SVG cells) that can support the replication of JCV at a slow rate [37] was superinfected with HHV-6. Superinfection of these JC-infected SVG cells with HHV-6 resulted in increased production of JCV infectious particles as measured by hemagglutination assay (Figure 4A). These results were supported by real-time PCR for the JCV-T antigen region (Figure 4B) and demonstrated that superinfection with HHV-6 resulted in increased JCV copy number over time compared to JCV-infected SVG cells alone. Moreover, *in situ* hybridization studies (Figure 4C) also demonstrated that superinfection with HHV-6 increased the percentage of SVG cells replicating JCV DNA.

## Discussion

The potential association of natalizumab treatment with reactivation of JCV and the subsequent development of PML is clearly a cause for concern. PML is a demyelinating brain disorders most often seen in setting of immunusuppression such as HIV infection and in transplant recipients. Natalizumab, an α_4_ integrin antagonist, blocks the binding of lymphocytes to adhesion molecules on the vascular endothelium, which could effectively decrease lymphocyte trafficking primarily to the gut and brain. It has been previously demonstrated that blocking α_4_ integrins can inhibit the transport of both CD4^+^ and CD8^+^ T cells to the CNS [38]. Given this mode of action of natalizumab and the potential consequence of PML in a subset of treated patients, it has been suggested that natalizumab treatment could result in a state of localized immune suppression in the CNS which could potentially lead to the reactivation of latent viruses [11]. Stüve et al. recently demonstrated that MS patients undergoing natalizumab therapy exhibited strikingly decreased CD4∶CD8 ratios in the CSF (comparable to those of an HIV-1-infected patient cohort) compared to MS patients not receiving natalizumab and they attributed this to decreased migration of CD4^+^ T cells into the CNS [10], [12]. Although the biological significance of this is unknown, the results are consistent with the hypothesis that natalizumab treatment may result in decreased immunosurveillance in the CNS, potentially allowing for the reactivation of latent viral infections. As further evidence of the ability of natalizumab to contribute to an immunocompromised state, our current results demonstrate a statistically significant decrease in the amount of total serum IgG in MS patients treated with natalizumab versus control cohorts not receiving this drug. Decreased serum IgG levels have long been associated with immunosuppression and opportunistic infections including viral reactivation [39], [40].

If the changes in normal immune function observed following natalizumab treatment result in decreased immune surveillance, we hypothesize that the potential for viral reactivation would not necessarily be limited to JCV. A number of other commensal human viruses maintain latency in the CNS including HHV-6 [41], which has been previously been found to be present in and around white matter lesions in PML patients [28]. Our data suggest that HHV-6 may be reactivated in MS patients receiving natalizumab therapy, as evidenced by the detection of viral DNA in the CSF of 2 out of 23 natalizumab-treated patients but not in samples from 16 untreated MS patients from the same institution. Since failure to detect HHV-6 in CSF is a consistent finding from untreated NIH MS patients and control patients with other neurologic diseases, it was therefore surprising to detect the presence of low levels of HHV-6 in MS patients treated with natalizumab. Cell-free HHV-6 DNA is suggestive of active HHV-6 infection while cell-associated DNA may be more commonly associated with viral latency [22]. Moreover, the low number of CSF samples from natalizumab-treated MS patients that tested positive for HHV-6 sequences, although higher than what was expected from untreated MS patients, may be a function of the limited size of this cohort; or alternatively HHV-6 in the CSF may not be reflective of the levels of HHV-6 in the CNS. We have recently reported that detection of CSF HHV-6 from patients with neurologic disease following bone marrow transplant did not correlate with the amount of HHV-6 that could be demonstrated in post-mortem brain sections [31]. Indeed, at times when the CSF was either negative for HHV-6 or from samples in which HHV-6 sequences could only be amplified by nested PCR, areas of the brain could have as many as 1 ×10^7^ copies/10^6^ cells as determined by quantitative PCR [31]. These observations suggest the use of caution in the interpretation of negative CSF results and may have application to other viruses such as JCV [6]. Additional support for the presence of HHV-6 in the natalizumab-treated MS patients came from HHV-6 variant-specific typing. HHV-6 viral DNA sequences obtained from the natalizumab-treated MS patients confirmed that these sequences were specific for the HHV-6A variant. These results are consistent with the reported tropism of HHV-6A, which has been more often associated with MS [18], [22], [25].

The detection of HHV-6 in natalizumab-treated MS patients was further supported by serologic findings demonstrating statistically higher levels of anti-HHV-6 IgG in serum from MS patients undergoing natalizumab therapy compared to controls. As there is no reliable commercially available serologic assay for the detection of HHV-6-specific antibodies, we developed and characterized a novel electrochemiluminescence- based methodology using HHV-6-infected cell lysates. This method was shown to be highly reproducible and specific. There was no statistically significant difference between MS serum anti-HHV-6 IgG and controls, consistent with our previous findings [24]. In contrast, natalizumab-treated MS patients had significantly higher levels of serum HHV-6 IgG than control, untreated MS patients. Since HHV-6 is a ubiquitous β-herpesvirus acquired early in life, it was not surprising to find that serum HHV-6 IgG is present in non-treated MS patients and falls in the empirically determined ‘normal range’ [24]. What was unexpected, given the limitations of such a small cohort of natalizumab treated MS patients, was the significant increase in the number of these patients determined to be above this normal range, both in the frequency of patients (11 of 23 natalizumab treated MS patients were above the normal range compared to only 1 of 17 untreated MS patients) and the magnitude of these responses (mean value of 5000 (range 0–10,000) units in the natalizumab treated MS patients compared to 1500 units (range 0–3000). Increased antibodies to HHV-6 could represent an immune response associated with a more recent exposure to this virus and would be consistent with the hypothesis that natalizumab treatment may be linked with viral reactivation.

Analysis of HHV-6 specific IgG in CSF was less informative since CSF HHV-6 IgG was elevated (p<0.0455) in all MS groups compared to CSF from control patients with OND. CSF from the OND cohort gave HHV-6 specific IgG values that were defined empirically as falling within a normal range (CSF from healthy controls were not obtained). The utility of this novel electrochemiluminescence assay was again validated using CSF from patients with encephalitis from whom a significant percentage (p  =  0.0110) was demonstrated to contain anti-HHV-6 IgG in the CSF that correlated with CSF PCR for HHV-6 DNA (manuscript in preparation). Increased CSF IgG to many viruses has been reported in MS patients and we are sensitive to the over-interpretation of what may be epiphenomenal results [42], [43]. However, there was a clear impression that in the CSF of a subset of natalizumab-treated MS patients there were higher levels of HHV-6-specific IgG compared to untreated MS patients, particularly those from the same center (Figure 3B), although this did not reach statistical significance. None of the natalizumab-treated MS patients that had positive CSF anti-HHV-6 IgG responses had positive serum HHV-6 IgG, suggesting that the elevated levels of HHV-6 specific IgG in CSF were not merely spillover from peripheral blood. These results clearly demonstrate the need to expand these observations to larger cohorts of natalizumab-treated and control patients in order to determine if increased intrathecal production of HHV-6 IgG is definitively associated with natalizumab treatment.

If natalizumab treatment is associated with decreased immune surveillance resulting in the reactivation of CNS viruses such as JCV [44] and HHV-6 (present report), can these two viruses be causally linked? The molecular mechanism by which JCV becomes reactivated in immunosuppressed individuals is unknown. Previously, Winklhofer et al. demonstrated that human cytomegalovirus (HCMV), another member of the beta- herpesvirus family, was able to transactivate JCV via the HCMV immediate-early transactivator 2 (IE2) protein, which stimulated JCV T antigen expression associated with JCV late gene expression [45]. Consistent with the hypothesis that HHV-6 may be associated with up regulation of JCV, previous studies have co-localized HHV-6 and JCV in PML lesions by double immunohistochemical techniques [28]. Our current work supports these results and we have also demonstrated the presence of HHV-6 in brain lesions of PML patients by immunohistochemisty for HHV-6 protein, real-time PCR for HHV-6 DNA, and sequence confirmation of HHV-6 (data not shown). Brain lesions of natalizumab-treated patients with PML were unavailable for analysis. The co-localization of HHV-6 in PML lesions suggested a possible mechanism whereby HHV-6 might be capable of reactivating JCV. This hypothesis was directly tested by infection of the persistently JCV-infected SVG human astrocytic cell line [37] with HHV-6. Infection of these JCV-SVG cells with HHV-6 resulted in increased production of JCV infectious particles as well as an increase in viral copy number measured by real-time PCR and an increase in the total number of cells replicating JCV DNA. These results suggest that HHV-6 infection could augment JCV replication and, in the setting of natalizumab treatment, may have important implications for JCV reactivation and the subsequent development of PML. In addition, as suggested for PML reactivation in natalizumab treated MS patients [46], [47], the blockade of a_4_ integrin could affect normal bone marrow physiology resulting in release of premature lymphoid progenitor harboring latent virus(es) into the circulation. HHV-6 has also been detected in CD34+ progenitor cells in both normal and diseases states [48], [49]. Collectively, these findings suggest that VLA4 antagonism may promote both JC and HHV-6 viral reactivation through impaired retention of CD34+ stem cells as well as impaired immune surveillance.

The small number of available samples from natalizumab-treated patients is an obvious limitation to this current study but, unfortunately, larger numbers of samples from additional cohorts are not available at this time. Re-approval of this drug by the FDA on June 5^th^ 2006 and by the regulatory agency of the European Union on June 29^th^ 2006 affords new opportunities to test the hypothesis that natalizumab treatment may be associated with an increase in latent viruses of the CNS. These studies are currently being planned in a multi-center trial with the Veterans Administration. However, even with the limitation of sample size, our present work in conjunction with previously published findings undoubtedly represents a need for caution as patients return to treatment with natalizumab. Prospective measures of immune function and viral reactivation should be initiated upon return to treatment in order to actively assess the risk of adverse reaction.

## Materials and Methods

### Patient Samples

Serum and CSF from 23 relapsing-remitting MS patients [50] treated with natalizumab (22 who received natalizumab in clinical trials, 1 patient who received natalizumab after its approval) and 17 MS patients who had not received natalizumab were obtained from the University of Texas Southwestern Medical Center at Dallas Multiple Sclerosis Center (Table 1). Serum and CSF samples from MS patients, healthy volunteers, and BMT patients were collected at the NIH Clinical Center. CSF from patients with encephalitis of unknown origin was obtained from the California Health Department. CSF was also obtained from 9 patients undergoing allogeneic BMT as described [31] and from patients with other neurologic diseases evaluated at the NIH clinical center (Table S1). Informed consent was obtained from all patients, and the respective Institutional Review Boards approved all study procedures.

Details on the patient cohort from UT Southwestern and Texas Neurology are published elsewhere [10], [12]. Patients received a median of 30 doses of natalizumab (range: 1–41). The median interval between the last natalizumab treatment and sampling of CSF and peripheral blood was 34 days (range: 5–45 days). The mean time of disease duration was 8 years (±3.8 years). The median time since last relapse from when the sample was taken was 2.63 years with a range from 0.17–3.04 years. The mean number of Gd-enhancing lesions was 0 (±0). The NIH RRMS group had a median disease duration of 4.9 years with a range from 2 months to 28 years. The median time since last relapse from when the sample was taken was 0.42 years with a range from 0–2.75 years. The mean number of Gd-enhancing lesions was 3(±6) with a range from 0–29.

### Detection of HHV-6 DNA and variant subtyping by sequencing

DNA was isolated using extraction kits according to manufacturers' instructions [51] and PCR amplified as described [52]. PCR products were purified using the Qiagen MinElute PCR Purification Kit (Qiagen, CA) and sequenced with Dye-Terminators (Perkin Elmer) for HHV-6 subtyping.

### Detection of anti-HHV-6 IgG and total human IgG

Antibodies against HHV-6 proteins were detected using electrochemiluminescence technology (MSD, Gaithersburg, MD) developed in our laboratory. Characterization of assay sensitivity and specificity are described in the supplemental information (Text S1, Figure S1). HHV-6B or mock-infected (SupT1) cell lysate prepared as described [25] was spotted onto plates (L17XB) and allowed to dry at room temperature (RT). 200 µl of Blocker A solution was added to each well and plates were blocked on a shaker for 1 hour at RTand then washed twice with 300 µl PBS. Serum/CSF were diluted in MSD Antibody Diluent and added to plates in 25 µl per well and incubated on shaker at RT for 1 hour and washed twice with PBS. Samples were tested in duplicate. 25 µl Sulfo-Tag™ labeled goat anti-human IgG (1 µg/ml) was added to each well, incubated and washed as described above. 150 µl MSD Read Buffer T was added to each well before analysis with a MSD PR400 plate reader. Total human IgG was measured in duplicate using the Easy-Titer® IgG Assay Kit (Pierce) according to the manufacture's protocol. The range of detection is between 15–300 ng/ml. Nonparameteric Mann-Whitney U-test was used for group analysis in this study to obtain a more conservative evaluation of statistical significance.

### HHV-6 infection of SVG cells

SVG cells [37], a human fetal astroglial cell line chronically infected with JCV (Mad-4 strain) were seeded onto 6 well plates overnight. Cells were washed twice with PBS followed by incubation for 3 hours at 37°C in 5% CO_2_ with cell-free HHV-6 supernatant. Cells were washed with PBS and replaced with medium. Cell culture JCV eplication was assessed by CPE, hemagglutination, in situ hybridization and quantitative PCR [53], [54], [55] and Mock infections were carried out using supernatant from uninfected SupT-1. Infections of each HHV-6 variant were performed twice.

## Supporting Information

Text S1 Characterization of a novel electrochemiluminescece assay for detection of HHV-6 using monoclonal anti-HHV-6 antibodies.
(0.04 MB DOC)

Figure S1 Characterization of a novel electrochemiluminescece assay for detection of HHV-6 using monoclonal anti-HHV-6 antibodies. (A) HHV-6B lysate (Z29 infected SupT-1 cells) reactivity with HHV-6 specific p41 monoclonal antibody. Reactivity is measured as photon emission. (B) HHV-6B lysate (Z29 infected SupT-1 cells) reactivity with larger panel of HHV-6 specific monoclonal antibodies except for the HHV-6A specific anti-p41/38 monoclonal antibody. (C) Lysate of uninfected SupT-1 cells reactive with the panel of HHV-6 specific antibodies. (D) Capture-detection assay using HHV-6 variant B lysate with the indicated monoclonal antibodies. (E) Capture-detection assay using HHV-6 variant A lysate with the indicated monoclonal antibodies. (F) Reactivity of 14 healthy control sera (dotted lines) against HHV-6B lysate. Solid black line illustrates the average antibody reactivity against HHV-6 in 14 healthy controls. (G) Sera from two natalizumab-treated patients TY20 and TY11 were tested for antibody reactivity against HHV-6B antigens at various dilutions as indicated. For panels D-G, reactivity is measured as photon emission on virus infected lysate minus photon emission on uninfected cells.
(1.55 MB TIF)

Table S1 List of other neurologic diseases
(0.03 MB DOC)
