# Supplementary material for: Reactivation of Human Herpesvirus-6 in Natalizumab Treated Multiple Sclerosis Patients
Source: PLoS One. 2008 Apr 30;3(4):e2028. doi: 10.1371/journal.pone.0002028 (PMC2323568; doi:10.1371/journal.pone.0002028)
Supplement: Text S1 — Characterization of a novel electrochemiluminescece assay for detection of HHV-6 using monoclonal anti-HHV-6 antibodies. (0.04 MB DOC) [file pone.0002028.s001.doc]

## Characterization of electrochemiluminescence assay for detection of anti-HHV-6 IgG

To support the hypothesis that HHV-6 may be reactivated in MS patients treated with natalizumab, screening of MS sera and CSF for the presence of anti-HHV-6 antibodies was performed. As there is a paucity of quantitative assays for the detection of anti-HHV-6 antibodies, a novel electrochemiluminescence assay was developed using HHV-6-infected cell lysates. Characterization of this assay is shown in figure S1. To assess the specificity of this assay, commercially available antibodies against HHV-6 proteins were first tested by comparing the binding of these antibodies to HHV-6 infected SupT-1 lysates versus uninfected SupT-1 cells. Figures S1 panel A and S1 panel B show that antibodies against the HHV-6 proteins p41, gp110 and 101K could clearly bind to HHV-6-infected cell lysates while no appreciable binding was observed when uninfected SupT-1 were used (Figure S1 panel C), with the anti-HHV-6 p41 antibody demonstrating the highest response (Figure S1 panel A). In a variation of this assay in which biotinylated anti-HHV-6 antibodies were first bound to streptavidin-coated wells as a means to “capture” the cell lysate, we were able to distinguish between the two variants of HHV-6. Antibodies that cross-react with HHV-6A and HHV-6B, anti-gp110 and anti-p41, reacted with both HHV-6B and HHV-6A lysates, whereas the HHV-6A-specific p41/38 antibody only reacted with the HHV-6A lysate (Figure S1 panels D and E).

Initial experiments to assess the ability of this assay to detect anti-HHV-6 IgG in normal human sera demonstrated that anti-HHV-6-specific IgG could be detected in a dose-dependent manner and differed between healthy donors (figure S1 panel F). This assay proved to be highly reproducible by testing the same samples on different days (data not shown). Two sera from natalizumab treated MS patients were initially tested and demonstrated anti-HHV-6 antibodies that again was dose-dependent and appeared to discriminate a high HHV-6 responder (TY20) from a lower response (TY11) (figure S1 panel G). This detection of HHV-6 specific antibody directly correlated with reactivity measured using a standard non-quantitative immunofluorescence method (data not shown). These experiments serve to characterize this novel HHV-6 ECL assay demonstrating a highly specific and robust method for the detection of anti-HHV-6 antibodies. Based on the results from normal sera (figure S1 panel F), a 1:200 and 1:400 dilution was operationally used in subsequent experiments to measure serum anti-HHV-6 antibodies.
