# Supplementary figures and images for: Reactivation of Human Herpesvirus-6 in Natalizumab Treated Multiple Sclerosis Patients
Source: PLoS One. 2008 Apr 30;3(4):e2028. doi: 10.1371/journal.pone.0002028 (PMC2323568; doi:10.1371/journal.pone.0002028)

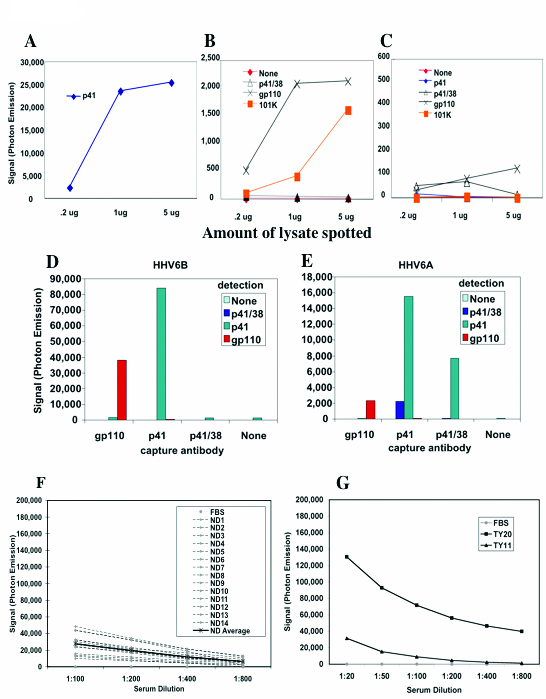

Supplement: Figure S1 — Characterization of a novel electrochemiluminescece assay for detection of HHV-6 using monoclonal anti-HHV-6 antibodies. (A) HHV-6B lysate (Z29 infected SupT-1 cells) reactivity with HHV-6 specific p41 monoclonal antibody. Reactivity is measured as photon emission. (B) HHV-6B lysate (Z29 infected SupT-1 cells) reactivity with larger panel of HHV-6 specific monoclonal antibodies except for the HHV-6A specific anti-p41/38 monoclonal antibody. (C) Lysate of uninfected SupT-1 cells reactive with the panel of HHV-6 specific antibodies. (D) Capture-detection assay using HHV-6 variant B lysate with the indicated monoclonal antibodies. (E) Capture-detection assay using HHV-6 variant A lysate with the indicated monoclonal antibodies. (F) Reactivity of 14 healthy control sera (dotted lines) against HHV-6B lysate. Solid black line illustrates the average antibody reactivity against HHV-6 in 14 healthy controls. (G) Sera from two natalizumab-treated patients TY20 and TY11 were tested for antibody reactivity against HHV-6B antigens at various dilutions as indicated. For panels D-G, reactivity is measured as photon emission on virus infected lysate minus photon emission on uninfected cells. (1.55 MB TIF) [file pone.0002028.s002.tif]
