# Supplementary material for: Reactivation of Human Herpesvirus-6 in Natalizumab Treated Multiple Sclerosis Patients
Source: PLoS One. 2008 Apr 30;3(4):e2028. doi: 10.1371/journal.pone.0002028 (PMC2323568; doi:10.1371/journal.pone.0002028)
Supplement: Table S1 — List of other neurologic diseases (0.03 MB DOC) [file pone.0002028.s003.doc]

Table S1. List of Other Neurologic Diseases Controls

| **Other Neurologic Diseases** | **Number of cases** |
| --- | --- |
|  |  |
| West Nile Virus encephalitis | 2 |
| Subacute sclerosing panencephalitis | 1 |
| SCID* | 1 |
| Epilepsy | 3 |
| Lupus | 1 |
| Encephalopathy post allogeneic BMT† | 1 |
| Encephalopathy after seizure | 1 |
| Sjögren’s | 1 |
| Encephalitis with leukemia | 3 |
| Vasculitis | 3 |

* Severe combined immunodeficiency; † Bone marrow transplant.
